# Cedrol, a malaria mosquito oviposition attractant is produced by fungi isolated from rhizomes of the grass *Cyperus rotundus*

**DOI:** 10.1186/s12936-016-1536-7

**Published:** 2016-09-17

**Authors:** Lynda K. Eneh, Hiromi Saijo, Anna-Karin Borg-Karlson, Jenny M. Lindh, Gunaratna Kuttuva Rajarao

**Affiliations:** 1 Chemical Ecology Unit, Division of Organic Chemistry, KTH Royal Institute of Technology, Stockholm, Sweden; 2 Forest Products Group, Faculty of Agriculture, Yamagata University, Tsuruoka, Japan; 3 Division of Industrial Biotechnology, School of Biotechnology, KTH Royal Institute of Technology, Stockholm, Sweden

**Keywords:** Malaria, Oviposition attractant, Fungi, Rhizome, Cedrol

## Abstract

**Background:**

Cedrol, a sesquiterpene alcohol, is the first identified oviposition attractant for African malaria vectors. Finding the natural source of this compound might help to elucidate why *Anopheles gambiae* and *Anopheles arabiensis* prefer to lay eggs in habitats containing it. Previous studies suggest that cedrol may be a fungal metabolite and the essential oil of grass rhizomes have been described to contain a high amount of different sesquiterpenes.

**Results:**

Rhizomes of the grass *Cyperus rotundus* were collected in a natural malaria mosquito breeding site. Two fungi were isolated from an aqueous infusion with these rhizomes. They were identified as *Fusarium falciforme* and a species in the *Fusarium fujikuroi* species complex. Volatile compounds were collected from the headspace above fungal cultures on Tenax traps which were analysed by gas chromatography–mass spectrometry (GCMS). Cedrol and a cedrol isomer were detected in the headspace above the *F. fujikuroi* culture, while only cedrol was detected above the *F. falciforme* culture.

**Conclusion:**

Cedrol an oviposition attractant for African malaria vectors is produced by two fungi species isolated from grass rhizomes collected from a natural mosquito breeding site.

## Background

Recently, the sesquiterpene alcohol cedrol was identified as an attractant for gravid female *Anopheles gambiae* mosquitoes that are vectors of the human malaria parasites [1]. The compound was detected as a volatile from an infusion made with water and soil taken from a natural *Anopheles* breeding site. A similar soil infusion had previously been shown to mediate an increased oviposition response in cage bioassays when incubated for 6 days compared to 2 and 4 days [2]. In addition, a 6 day old aqueous infusion with soil from the same site was shown to attract *An. gambiae* females to gravid traps in semi-field settings [3]. Furthermore, cedrol was detected in higher amount from a non-autoclaved soil infusion compared to the same infusion that had been autoclaved [1]. These results suggested a possible microbial link and together with reports about sesquiterpenes as common fungal metabolites [4, 5] indicates that the cedrol released from the soil infusion may be of fungal origin.

*Cyperus rotundus* grass, also known as nut grass, was found growing in the natural *Anopheles* breeding site where the soil was collected in the previous studies [1–3]. Furthermore, essential oil from rhizomes of this grass has been reported to contain high amounts of sesquiterpenes [6] and cedrol has been found in rhizome extract of another grass species in the same genus, *Cyperus articulates* [7]. Based on these studies it was hypothesized that fungi associated with *C. rotundus* rhizomes may be the source of the oviposition attractant cedrol. To test this, fungi were isolated from rhizomes of *C. rotundus* grass collected from the same natural *Anopheles* breeding site used for the soil infusion studies described above. Furthermore, volatiles were collected from the headspace above cultures of these fungi and analysed on a gas chromatograph coupled to a mass spectrometer.

## Methods

### Fungi isolation and identification

**Fig. 1:**
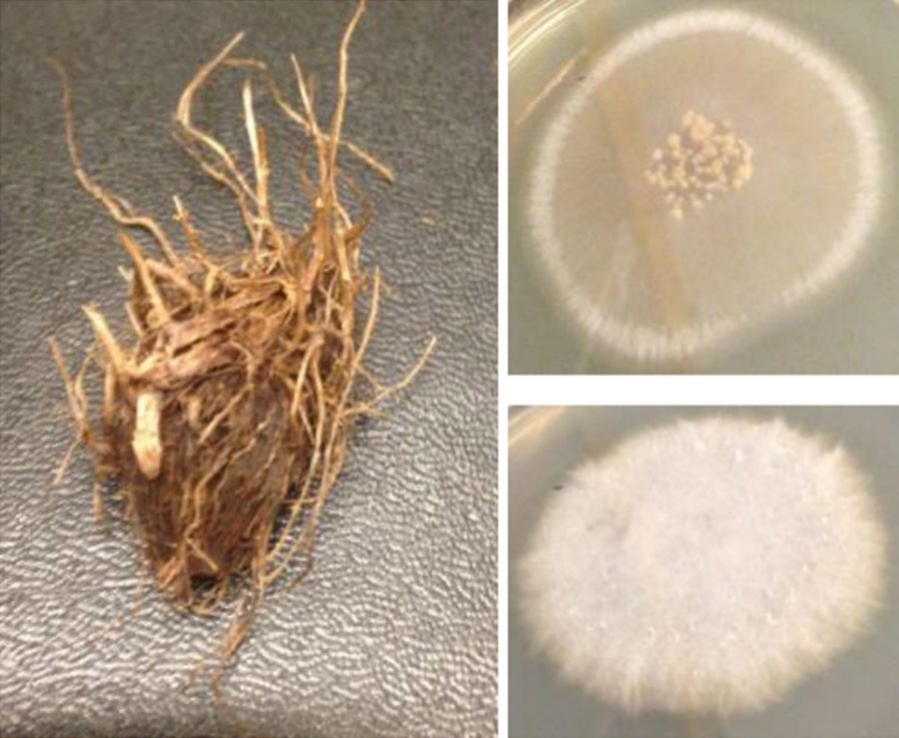
Rhizome of *Cyperus rotundus* and the two fungal cultures; *top*: *F. falciforme* Carrion, *below*: a species in the *F. fujikuroi species* complex

Soil with *C. rotundus* rhizomes (Fig. 1) was collected from a malaria mosquito breeding site at the international centre of insect physiology and ecology—the Thomas Odhiambo campus in Mbita, Western Kenya—and transported to Sweden. The rhizomes were harvested and washed in sterile water five times to remove any traces of soil and then incubated in sterile water at a concentration of 70 g/l (rhizome-water infusion). The infusion was allowed to stand for 2 days (R2D) or for 6 days (R6D) at room temperature (22 ± 2 °C). Samples of the rhizome infusion (100 µl) were spread on YEG agar plates, which were incubated at room temperature for 2–6 days.

Yeast extract glucose (YEG) agar was prepared by adding 4 g of yeast extract (Applichem: Chemtronica AB, Stockholm Sweden), 4 g of d-glucose (Applichem; Chemtronica AB, Stockholm Sweden) and 15 g agar (Fluka analytical, Sigma, Stockholm Sweden) to 1 L of deionized water. This mixture was sterilized using an autoclave at 121 °C for 20 min. YEG broth was prepared the same way but without the agar.

Single isolates from the plates were transferred to new YEG agar plates and incubated at room temperature for 2–6 days and repeated sub-culturing until single isolates were obtained and confirmed by observation under a microscope. The isolates were sent to CBS-KNAW Fungal Biodiversity Centre for identification (Centraalbureau voor Schimmelcultures, Netherlands).

### Headspace collections from fungal cultures

The fungal isolates were inoculated into separate E-flasks (500 ml) with 300 ml YEG broth. Headspace samples were collected on Tenax traps from the headspace above fungal cultures after incubation for 6 days. The E-flask was fitted with a gas wash bottle head (QuickFit joined ware, Staffordshire, United Kingdom) for volatile collections. The traps were made from 25 mg of Tenax^®^ TA polymer (60–80 mesh, Supelco, Bellefonte, PA, USA), placed in GERSTEL-Twister Desorption glass liners (GERSTEL, Muelheim an der Ruhr, Germany). The polymer was held in place with glass wool (Supelco, Bellefonte, PA, USA) on each side. The traps were washed 10 times with 200 µl of methyl tertbutyl ether (MTBE) and sealed with polytetrafluorethylene (PTFE) tape and placed in oven at 40 °C for 4 h before use. Charcoal filtered air was pumped into the E-flasks at a rate of 0.1 l/min and drawn out at the same rate through a Tenax trap. The traps were sealed with PTFE tape after collection and stored at −80 °C. E-flasks containing 300 ml of sterile YEG agar and empty E-flasks were sampled the same way and utilized as controls for culture medium and Tenax trap background volatiles.

### Gas chromatography–mass spectrometry (GC–MS) analysis

Tenax traps with fungal headspace collections were analysed on an Agilent 7890A gas chromatograph connected to an Agilent 5975C inert MSD with Triple Axis detector mass spectrometer (Agilent, Santa Clara CA, USA). The GCMS system was fitted with a GERSTEL Multi-Purpose Sampler (MPS: Gerstel GmbH & Co. KG, Mülheim an der Ruhr, Germany). The GC capillary column utilized was an Agilent’s HP-5MS column (5 % phenyl and 95 % dimethyl polysiloxane, 30 m, 250 µm internal diameter and 0.25 µm film thickness). Prior to analysis one microliter heptyl acetate (3.16 ng/µl) was added to the Tenax trap in the GERSTEL thermal desorption unit (TDU) followed by thermal desorption of traps in splitless mode at an initial temperature of 40 °C which was then increased by 120 °C/min to 270 °C, the end temperature was held for 5 min. The desorbed volatiles were focused in a GERSTEL CIS inlet at 10 °C. The CIS inlet operated in splitless mode was then heated at a rate of 12 °C/s to 280 °C during which the volatiles were transferred to the column. The MS at full scan identified mass ranges from 30 to 400 m/z with electron ionization at 70 eV and ion source temperature at 230 °C.

The GC–MS data were analysed with Agilent’s enhanced Chemstation software version E.02.01.1177. The data were screened for two of the main MS ions of cedrol (95, 150). The identified peak was confirmed to be cedrol by comparing the retention time and mass spectra to a sample of an authentic standard of cedrol [(+)-cedrol, ≥99.0 % sum of enantiomers, GC, optical activity α_D_^20^ +10.5 ± 1°, Sigma-Aldrich Sweden AB, Stockholm, Sweden] analysed using the same GCMS settings as described above. Epi-cedrol and α-acorenol were identified based on comparison of the mass spectra to mass spectra in the NIST 08 library and in Brock et al. [8].

## Results and discussion

**Fig. 2:**
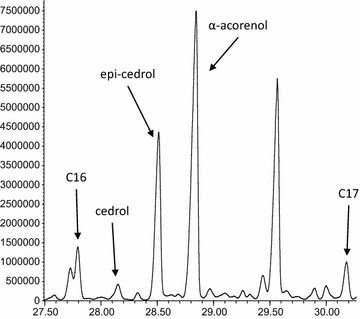
Cedrol, epi-cedrol and a-acorenol detected in the headspace above a *F. fujikuroi* culture

Two fungi were isolated from the aqueous rhizome infusion and identified as a species in the *Fusarium fujikuroi* species complex and *Fusarium falciforme* (Fig. 1). *Fusarium* is a large genus of filamentous fungi that are widely distributed in soil, often associated with plants. *F. fujikuroi* species can be pathogens for rice [9] and cause bakanae disease (“foolish seedling disease”) through metabolism of high amounts of gibberellins, which are plant hormones that promotes growth. A fungus in the *F. fujikuroi* species complex has previously been reported to produce a large range of volatile compounds where the majority were sesqui- and diterpenes, including well known precursors of gibberellins [8, 10]. The main sesquiterpene was identified as a-acorenol, however also small amounts of cedrol and epi-cedrol were detected [8, 10]. All three of these compounds were also detected as metabolites of the *F. fujikuroi* isolate included in this study (Fig. 2) while only cedrol was detected above the *F. falciforme* culture. It is possible that a longer incubation time or modified culture conditions might enhance the production of cedrol by these fungal species. It would be interesting to test the response of *An. gambiae* mosquitoes to these compounds and fungal cultures in oviposition bioassays [11]. Cedrol is well known as a component of essential oils of *Cupressus* and *Juniperus* species and have been found in a variety of other plants [7, 12–14]. Further studies are needed to elucidate if fungi associated with the plants are involved in the metabolism of the compound for any of these species.

Other fungi such as *Beauveria bassiana* have been shown to produce spores that are attractive to host seeking *Anopheles stephensi* mosquitoes [15]. This agrees with this study that fungi can produce cues that attract mosquitoes. In fact, fungal volatiles and insect orientation may be a common ecological phenomenon, for instance it has been observed that volatiles like 2-phenylethanol and 2-methyl-1-butanol from *Aureobasidium pullulans* are attractive to different insect taxa [16]. Furthermore, volatiles from several fungi were tested as oviposition attractants of female yellow peach moths and the result suggested that the compounds can be used in gravid traps to monitor oviposition [17].

## Conclusion

Cedrol, the first identified oviposition attractant for *An. gambiae s.s.* and *An. arabiensis* females was identified as a metabolite of two fungi species in the *Fusarium* genus. These fungi were isolated from *C. rotundus* rhizomes collected from a natural *Anopheles* oviposition site. This finding may be utilized in future studies to understand why malaria mosquitoes utilize cedrol as an attractant and how the compound and possibly the grass species may influence malaria mosquito ecology. Such knowledge could be utilized in development of novel control strategies targeting gravid females.
